# COVID-19 unemployment and access to statin medications in the United States

**DOI:** 10.3389/fpubh.2023.1124151

**Published:** 2023-03-30

**Authors:** Manuel Hermosilla, Caleb Alexander, Dan Polsky

**Affiliations:** ^1^Carey Business School, Johns Hopkins University, Baltimore, MD, United States; ^2^Bloomberg School of Public Health, Johns Hopkins University, Baltimore, MD, United States; ^3^School of Medicine, Johns Hopkins University, Baltimore, MD, United States

**Keywords:** statins, healthcare access, COIVD-19, unemployment, Medicaid, I13, I14, E34.

## Abstract

**Objective:**

To quantify the effect of the unemployment created by COVID-19 on access to (sales of) statin drugs in the United States population.

**Methods:**

Approximately half a billion transactions for statin drugs in the United States between January 2018 and September 2020 are analyzed. We studied the potential causal relation between abnormal levels of unemployment during the first wave of COVID-19 in the U.S. and abnormal levels of sales of statin products (both variables defined at the state/week level). Variables are analyzed using the Two-Stage Least Squares (2SLS) method, which exploits comparisons of statin sales between states where, given the occupational distribution of their workforce, unemployment was more structurally vulnerable to mobility restrictions derived from COVID-19 against states where it was less structurally vulnerable.

**Results:**

While we do not find unemployment effects on statin sales on most of the population, our estimates link COVID-fueled unemployment with a sharp sales reduction among Medicaid-insured populations, particularly those in working age. For the period between March and August of 2020, these estimates imply a 31% drop of statin sales among this population.

**Discussion:**

COVID-fueled unemployment may have had a negative and significant effect on access to statin populations among Medicaid-insured populations.

## 1. Introduction

As of June of 2022, the COVID-19 pandemic has resulted in over one million deaths by infection in the United States.^1^ However, the pandemic's effects on the health of the American population have far exceed those reflected by these deaths. The emergency has also created massive disruptions in access to health care, due to voluntary cancellations, postponements, and overstretched health systems (1, 2). At the height of the crisis, two in five individuals in the U.S. reported avoiding or delaying regular (non-COVID) care and one in five households reported facing difficulties in obtaining care for serious medical issues (3). Among other detrimental consequences, forgone care has translated into lower rates of vaccinations and routine check-ups among children (4, 5), as well as a reduction of preventive cancer screenings and newly diagnosed cancers among adults (6, 7). As a result, experts predict a long-term deterioration of health outcomes, particularly among vulnerable groups (8). In the U.S., this deterioration may already be perceptible in the form of excess non-COVID mortality, particularly from chronic conditions such as hypertension and heart disease (9).

These disruptions and the corresponding worsening of health outcomes have been exacerbated by the economic recession triggered by the pandemic, which fueled unemployment beyond the levels observed during the great recession (10). The large unemployment levels provoked by COVID-19 have been linked, for example, to greater psychological distress (11), higher rates of food insufficiency (12), and a deterioration of patients' health-related quality of life (13).^2^ To our knowledge, published research has not yet investigated whether the deteriorated economic conditions introduced by the pandemic may have also hindered access to essential medicines.

We examine the relationship between unemployment and access to statin medicines during the initial phase of the COVID-19 pandemic in the United States. Statins are a class of lipid-lowering pharmaceutical agents which play a central role in the treatment and prevention of coronary heart disease. As such, the class is a WHO-designated essential medicine and is currently used by over 80 million U.S. adults. Empirically, we analyze the relationship between longitudinal year-to-year changes (2020 vs. 2019) in statin sales at the state/week level against analog unemployment changes. We derive identification by exploiting structural labor market differences across states, which determined how vulnerable each state's unemployment levels were to the mobility restrictions created by the pandemic. For example, compared to Washington DC, a much larger share of Wyoming's workforce is employed in occupations that involve the operation of machinery, which cannot be performed from home. Accordingly, the same amount of COVID-fueled mobility reductions led to over twice as much unemployment in Wyoming than in Washington DC. By systematizing these structural labor market differences across states, we are able to incorporate this source of variation into a Two Stage Least Squares Instrumental Variables procedure.^3^

For estimation, we leverage statin sales data from the healthcare intelligence company IQVIA, which account for over 90% of U.S. retail sales in the U.S. The estimation sample includes data from February to August of 2020 (COVID-19's first wave), which includes the period where COVID-fueled unemployment peaked at levels not observed since the great recession. Our estimates reveal no unemployment effects on statin sales to privately- or Medicare Part D-insured populations, which constitute the majority of the sample (close to 95% of dispensed doses). However, we find large unemployment effects within the Medicaid population, which commands a small share of sales in our data. In particular, for the period between March and August of 2020, our estimates link COVID-fueled unemployment with a 31% drop of statin sales among this population. These effects, which are more pronounced among working age populations, do not appear to be driven by reduced access to medical appointments or by price changes. In all, we interpret these results as additional evidence of COVID-fueled disruptions to health care access in the U.S., particularly among disadvantaged populations.

The remainder of the paper is organized as follows. In Section 2, we describe the institutional and literature background. In Section 3, we describe the data used in the analysis and discuss our empirical strategy in Section 4. In Section 5 we present our main results, which include a series of robustness checks. In Section 6, we discuss our findings and draw policy implications. We then conclude with a brief summary in Section 7.

## 2. Background

### 2.1. The relationship between unemployment and health

Unemployment can impact human health through the large financial and emotional distress that it imposes on workers and their households. Prior research has documented this possibility, for example, through cases of diminished drug sales in areas of higher unemployment (18) and through cases of massive layoffs followed by excess mortality from heart conditions, suicide, self-harm, and alcoholism (19–21). At the same time, unemployment has been found to lead to the adoption of healthy behaviors and ultimately better health outcomes (22). This can occur, for example, because the increased availability of time fosters time-intensive health investments such as physical activity, diet, and the seeking of preventive medical care (23).

The empirical evidence of these two countervailing effects highlights that the effect of unemployment on health may be positive or negative. Beyond this ambiguity in the literature, recent developments in the United States warrant further study of this relationship. First, the Affordable Care Act has led to increased rates of health insurance coverage (24). As a result, unemployment may have become less financially consequential in relation to healthcare access and health outcomes. Second, the rise of the non-traditional work arrangements from the gig economy has endowed many workers with a flexible work schedule and, most importantly, with an accessible source of income when formal employment is lost (25). Lastly, the COVID-19 pandemic has prompted many American workers to reconsider their stance on work/life balance, suggesting the possibility of unemployment spells becoming less disruptive for workers' lives and subsequent capacity to maintain individual health.

### 2.2. Statin underutilization in the United States

Statins drugs are used to prevent the excess accumulation of cholesterol in the bloodstream, which is an important risk factor for cardiovascular events such as heart attacks and strokes. They operate by hindering the production of cholesterol by the liver.

In addition to being generally safe, statins are regarded as highly effective. For example, data from the Heart Protection Study (26) reveals that a regular regime of simvastatin (a leading statin product) significantly reduces the risk for cardiovascular events. Among people aged 70 years and older, this reduction implies between 0.47 and 0.76 additional life years. The increase in life expectancy is generally larger among people aged 40–49, i.e., between 0.71 and 1.33 additional life years.

Combined with the relatively high hospitalization costs and low prices for generic statin products, the reduced probability of cardiovascular events makes statin medications highly cost effective for U.S. patients. Based on Health Protection Study data, Grennan et al. (27) describe these benefits as a “flow” of dollarized health benefits amounting to about $500 per patient/year. Importantly, these gains materialize partly as system-wide savings in the form of avoided hospitalization costs (26). Given these benefits, there is general agreement that statin drugs are currently underutilized in the U.S. (27, 28).

## 3. Data

### 3.1. Statin sales

We obtained access to data on U.S. transactions for statin drugs collected by IQVIA's Longitudinal Prescription Claims database. IQVIA is a large healthcare intelligence company (formerly IMS Health) that each year processes about four billion claims for prescription drugs purchased in the U.S., accounting for over 90% of retail sales, 60–85% of mail service sales, and 75–80% of long-term care sales.

The sample starts in January of 2018 and ends in August of 2020. The file contains information on approximately half a billion transactions encompassing over 30 billion dispensed doses. Each record includes a product identifier (there are 20 different statin products), a transaction date, a transaction location (5-digit zip code of prescribing provider), a patient-level identifier and age group, and the number of (daily) doses included in the purchase. The data also include information regarding the type of insurance, in the form of a “pay type description” variable. This variable associates each transaction with one of four payment modalities: Cash, Medicaid, Medicare Part D, and Third Party.^4^ Together, Medicare Part D and Third Party transactions account for about 95% of all dispensed doses (in roughly equal parts). Cash transactions account for 2.3%; Medicaid transactions, for 1.1%.

Table 1 presents statistics on total dispensed doses by year. Since our empirical analysis is based on year-to-year differences between 2020 and 2019 sales, the sample restricts attention to doses dispensed between January and August of each year. The figures (millions of doses) highlight the small relative size of the segment of transactions paid with cash, and to that paid through Medicaid. They also reveal that, expect for the cash segment, the market exhibited a moderate amount of year-to-year growth (about 5%). For each payment type, Table 2 shows the fraction of sales associated to each of the listed age groups: less than 40 years of age, between 40 and 64 years, and 65+ years. For all payment modalities, purchases by people less than 40 years of age comprise the smallest share. Whereas, Medicare Part D transactions are dominated by purchases from people 65 years and older, Medicaid transactions are driven by people in the 40–64 range. For Cash and Third Party transactions, the split is more even between these two age groups, with an advantage for individuals aged 40–64 years old.

**Table 1:** Statin sales in 2019 and 2020 (January–August, in millions of daily doses).

**Payment type**	**January'19 –Augus'19**	**January'20 –August'20**
Cash	221	186
Medicaid	95	98
Medicare Part D	4,049	4,311
Third Party	4,004	4,166

The table reports the total number of statin doses dispensed during January-August of each year, in millions. The data source is IQVIA's Longitudinal Prescription Claims database.

**Table 2:** Statin sales by age group.

**Payment type**	** < 40**	**40–64**	**65+**
Cash	0.04	0.52	0.43
Medicaid	0.08	0.72	0.20
Medicare Part D	0.01	0.09	0.90
Third Party	0.04	0.64	0.33

Shares of sales by age group, all row sums equal one.

### 3.2. Unemployment

**Figure 1:**
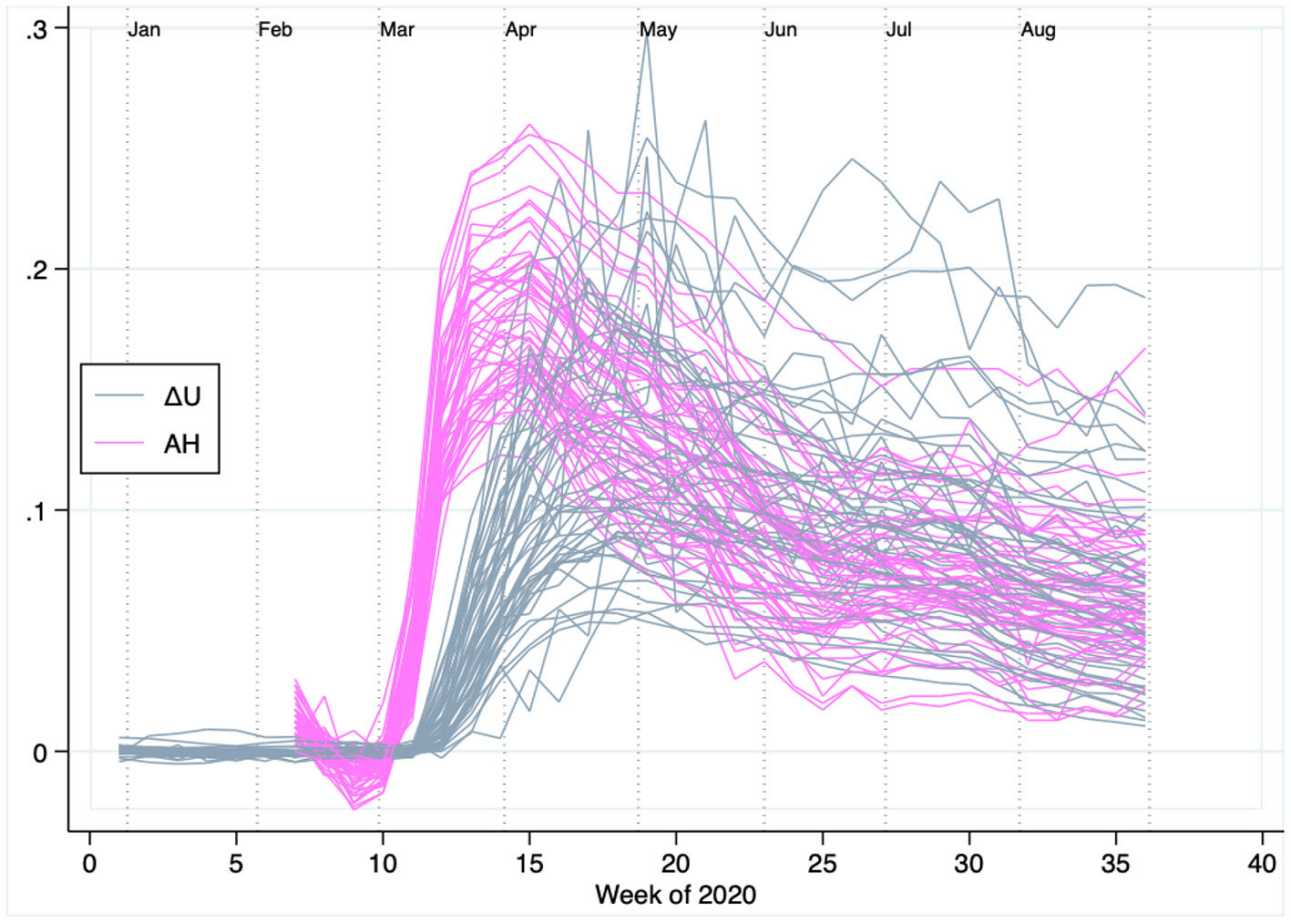
Abnormally high unemployment and low mobility in the United States during the first 36 weeks of 2020. Each pink line represents a state's year-to-year change in unemployment (Δ*U*) in 2020 vs. 2019. The flat pattern seen until the 12th week of 2020 (mid-March) indicates that, before shutdowns, unemployment rates across states were much like in the weeks of 2019. Each gray line represents abnormal stay-at-home rates, measured relative to the first six weeks of 2020. Consistent with a surge of unemployment fueled by mobility restrictions, the surge of stay-at-home rates precedes that of unemployment.

From the U.S. Department of Labor website, we obtained rates of insured unemployment (U). These series, which are available at the week/state level, are described by the blue curves of Figure 1 in the form of year-to-year differences or “abnormal unemployment.” In particular, for each state *i* and week of the year *t*, we plot $\Delta U_{it}=U_{it}^{2020}-U_{it}^{2019}.$ For example, Δ*U* = 0.07 would mean that the 2020 unemployment rate was seven percentage points larger than in the same state and week of 2019. This was the case for Maryland during the 15th week of 2020, when it experienced 8% unemployment rate compared to 1% during the same week of 2019. Until about the 12th week of 2020 (mid-March), before shutdowns, Δ*U* series display generally flat patterns. This suggests that, until then, states were experiencing similar unemployment rates in 2020 as compared to 2019. The subsequent evolution describes a large surge of unemployment. During April and May of 2020, states averaged 13 points of abnormal unemployment, whereas the average was about 10 points between June and August.

### 3.3. Mobility

We retrieved mobility data from Google's COVID-19 Community Mobility Reports. Based on the movement of consenting users of Google devices, these data track population mobility by computing the amount of time individuals spend in different types of locations. From these data, we retain the “stay-at-home” series, which track the amount of time individuals were connected to their home Wi-Fi network. As such, these series provide a summary for overall mobility, with lower mobility reflected by higher stay-at-home (AH) rates. The series are available starting from the 7th week of 2020, and expressed as differences relative to AH rates during the first 6 weeks of 2020. The purple lines of Figure 1 show state-level AH rates through the covered period. Consistent with the chronology of lockdowns, AH rates were relatively flat until mid-March (i.e., similar to as in the first 6 weeks of the year). Mobility was restricted in most places over the ensuing weeks, causing a surge of AH rates across all states. Consistent with the idea that mobility restrictions played a large role behind the COVID-19 economic downturn, the surge of AH rates preceded the surge of abnormal unemployment.

### 3.4. State teleworkability

As described in the next section, our empirical strategy relies on comparing statin sales between states where, given the occupational distribution, unemployment was more vulnerable to decreased mobility against states where employment was less vulnerable. We implement this comparison by relying on a state-level measure of “teleworkability,” i.e., the ease with which jobs can transition from being performed in the workplace to being performed at home. Our procedures closely follow the frameworks of Dingel and Neiman (16) and Mongey et al. (17), with minor modifications to fit our context.

In particular, we construct a state-level “inverse teleworkability score” (*ITW*), which ranges from 0 (maximum teleworkability) to 1 (minimum teleworkability). To do so, we combine two sources of data. First, we use the Occupational Information Network (O*NET) Work Context module survey to elicit how difficult each occupation is to be performed at home.^5^ We rely on the survey's “work context” and “work activities” modules, which ask about the importance of a series of activities that relate to teleworkability, for about 900 occupations (defined as 3-digit SOC codes). For example, the context module asks about the importance of email use while the activities module asks about the importance of physical activity. Jobs for which email use is more important or physical activity is less important are interpreted as being more teleworkable.

**Figure 2:**
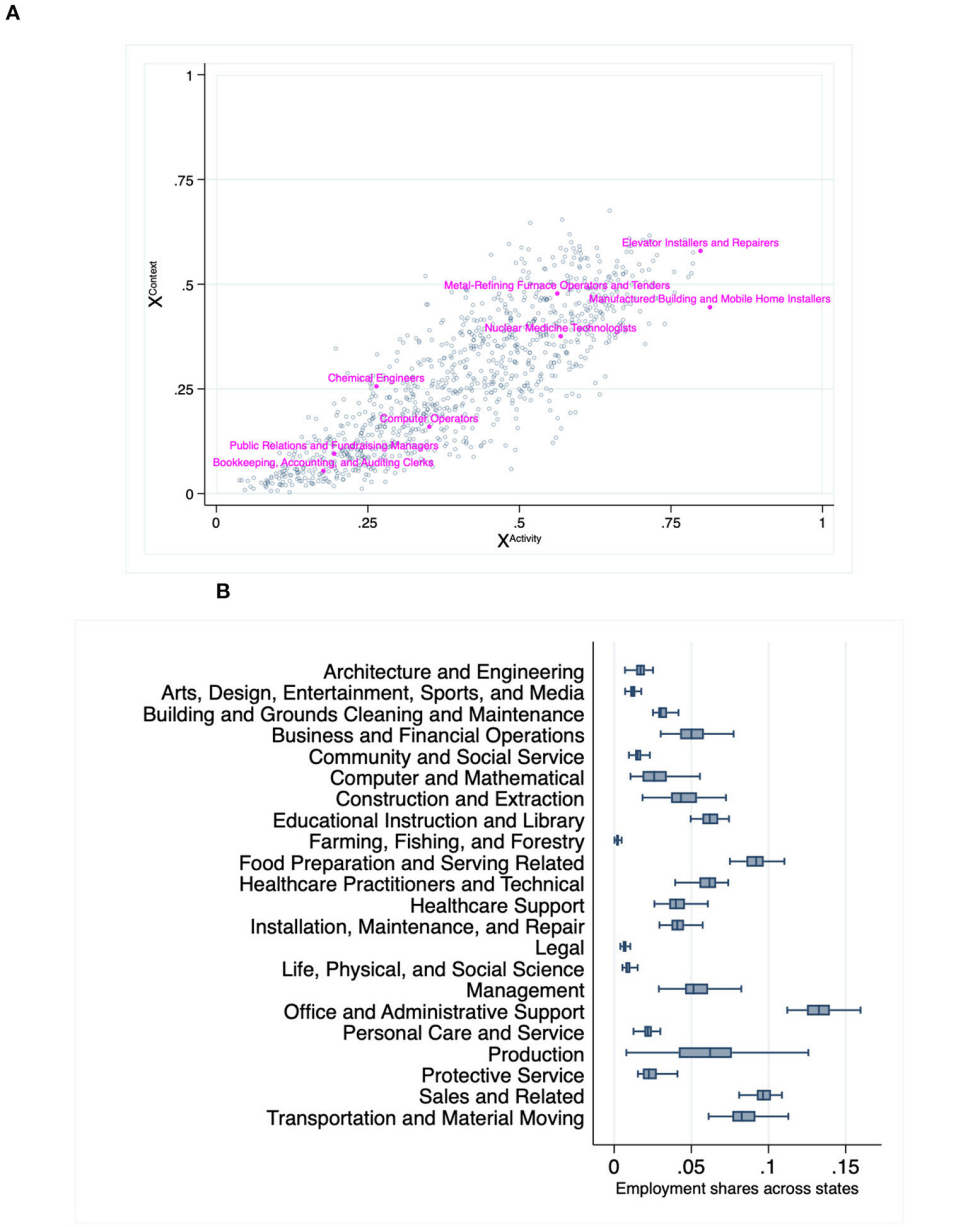
Elements of State-level Teleworkability. **(A)** Occupations' context and activity scores (lower scores reflect higher teleworkability). **(B)** Sectoral employment variability across states. **(A)** Data source: “Work Context” and “Work Activity” modules of the O*NET survey (https://www.onetonline.org/help/online/data). **(B)** Data source: BLS' Occupational Employment Statistics Survey (https://www.bls.gov/oes/tables.htm).

The survey includes 7 work context and 8 work activity questions, all of which are scored between 1 (low importance) and 5 (high importance).^6^ We normalize these scores to the unit interval and, when necessary, reverse the scores to ensure that lower values point to higher teleworkability for all questions. For each occupation o∈O, we summarize these data through pairs ${\left\{\left(x_{o}^{\text{Activity}},x_{o}^{\text{Context}}\right)\right\}}_{o\in O}$, where $x_{o}^{m}$ is the average score across all module *m* questions. Figure 2A shows these pairs across occupations. The figure shows that context and activity scores exhibit a strong positive correlation. The scores are also overall quite intuitive (for illustration, labels are added to a random set of occupations). For example, the occupation “Elevator Installers and Repairers,” which can hardly be performed from home, gets both high context and high activity scores (i.e., little teleworkability). In the other extreme, the occupation “Bookkeeping, Accounting and Auditing Clerks,” which seems well suited for work-from-home, receives relatively low scores.

The second data source corresponds to Occupational Employment Statistics Survey (OES), which is maintained by the Bureau of Labor Statistics.^7^ These data provide the distributions of each state's workforce across occupations. We express these distributions through weights ω_*io*_∈[0, 1] representing the share of state *i*'s 2019 active workforce employed in occupation *o*. Figure 2B shows that there exists significant cross-state variation in terms of the how important each occupation is within each state.^8^

With these two elements, we compute the state *i*'s inverse teleworkability score (ITW) as:


(1) $$\begin{array}{l} ITW_{i}=\underset{o}{\sum }\omega_{io}\cdot \frac{1}{2}\cdot \left(x_{o}^{\text{Activity}}+x_{o}^{\text{Context}}\right) \end{array}$$

The resulting distribution of *ITW* scores (presented in Table A.1 of the Appendix). The states receiving the five lowest ITW scores are Maryland, Connecticut, New York, Massachusetts, and Washington DC.^9^ Those receiving the five highest scores are West Virginia, Mississippi, North Dakota, Louisiana, and Wyoming. This variation is intuitive to us, in the sense that it coincides with our priors for where telework-friendly are more and less common.

## 4. Empirical strategy

### 4.1. Ordinary least squares regression

We seek to estimate the effect of unemployment on the sales (daily doses) of statin product *j* during the *t*^*th*^ week of 2020 in state *i*. To estimate this effect, a basic challenge stems from the presence of unobserved differences in baseline demand levels. For example, states with a larger elderly population may exhibit persistently higher demand for all statin products. Demand levels may also exhibit seasonality effects, for example, if adherence falls during summer-time vacations. To further complicate the matter, such persistent differences can be product specific. For example, patients may be more likely to “skip a pill” when they use a more expensive drug product (29).

To account for such effects, we formulate a regression that exploits the variation of abnormal 2020 sales, $\Delta Q_{ijt}=\log\left(1+Q_{ijt}^{2020}\right)-\log\left(1+Q_{ijt}^{2019}\right)$, where $Q_{ijt}^{Y}$ represents the total number of doses of product *j* sold in state *i* during week *t* of year *Y*, and where a 1 is added to avoid indefinition when *Q* = 0. For example, $Q_{\text{MD},j,2020}^{2020}$ and $Q_{\text{MD},j,2019}^{2019}$ represent the number of daily doses sold in Maryland, respectively during the 15th week of 2020 and the 15th week of 2019. As such, Δ*Q*_*jit*_ does not include the product/state/week component of demand that is common across both years.

Using data for weeks 7-36 (maximum window for which all variables are available), we estimate the following equation:


(2) $$\begin{array}{l} \Delta Q_{ijt}=\beta_{0}+\beta_{1}\Delta U_{it}+\beta_{2}AH_{it}+\mu_{i}+\theta_{j}+\lambda_{t}+\epsilon_{ijt}, \end{array}$$

The key regressor in this equation is Δ*U*_*it*_, which represents abnormal (year-to-year) unemployment in state *i* during week *t*, as discussed in Section 3.3 and plotted in Figure 1. As formulated, this key regressor is also deprived of the state/week amount of unemployment that is common across both years. An estimate $\hat{\beta_{1}}<0$ would associate abnormally high 2020 unemployment with abnormally low statin sales.

To avoid confounds, we endow (Equation 2) with a series of fixed effect controls. First, we include week-level fixed effects (λ). These aim to control for the effects of nation-wide policies rolled out during the pandemic which relaxed households' budget constrains (e.g., federal stimulus checks). We also introduce product-level fixed effects (θ), which are helpful to control, e.g., for product-lifecycle effects (e.g., brand name products with more generic competition may spend less on advertising). Lastly, we include state-level fixed effects (μ). These effects are used to control for possible state-level differences in access to healthcare (e.g., Medicare Advantage) and any interstate variance in unemployment compensation schemes. In addition to these three sets of fixed effects, Equation (2) controls for mobility levels through the stay-at-home rate AH, which varies at the state/week level (Figure 1). The term ϵ represents an error, which we cluster at the state level. Table 3 presents descriptive statistics for the main variables entering (Equation 2).

**Table 3:** Descriptive statistics of main variables.

**(1)**	**(2)**	**(3)**	**(4)**	**(5)**	**(6)**	**(7)**	**(8)**
**Variable**	**Notation**	**Level of variation**	***N***	**Mean**	**Std. Dev**.	**Min**	**Max**
Abnormal (log) statin sales	ΔQ	Product/State/Week					
Cash			30,600	-0.06	1.17	-6.77	6.66
Medicaid			30,600	-0.06	1	-6.97	8.77
Medicare Part D			30,600	0.03	1.41	-6.45	7.27
Third Party			30,600	-0.06	1.34	-6.35	6.18
Abnormal Unemployment	ΔU	State/Week	1,530	0.08	0.05	-0.01	0.3
At-home-rate	AH	State/Week	1,530	0.09	0.06	-0.02	0.26
Inverse Teleworkability Score	ITW	State	51	0.69	0.14	0	1 0

A noteworthy aspect of from Equation (2) is that it does not include a price control. This exclusion is motivated by the fact that price information is sometimes missing, specifically, in cases when *Q* = 0. However, the following reasons suggest that this exclusion should not be problematic for our inference. First, our equation controls for cross-sectional price variation through the inclusion of product-specific fixed effects. In turn, product-specific temporal price changes (2020 vs. 2019) operating nationwide are controlled for by the product-level fixed effects. Accordingly, price information missing from Equation (2) would bias our key estimate only if its longitudinal variation correlated with the evolution of abnormal unemployment. Leveraging the limited price information available from the data, we will present evidence suggesting that such bias is unlikely to be present in our estimates.

### 4.2. Two stage least Squares regression

An important caveat about the β_1_ estimate of Equation (2) is that, if the error term ϵ is correlated with the employment regressor, the estimate may not represent a causal relationship between abnormal unemployment and statin sales. This would be the case, for example, if those states that were more heavily hit by the pandemic (experiencing higher abnormal unemployment) also provided greater supplemental aids to their populations. Effects like this would lead to the underestimation of the causal impact of unemployment on statin sales. To guard against this possibility, we employ a Two Stage Least Squares (2SLS) instrumental variables procedure.

The essential component of the 2SLS procedure is to determine an instrumental variable (IV), i.e., a source of variation of abnormal unemployment that is plausibly uncorrelated with the error term of Equation (2). We construct the IV by exploiting states' teleworkability differences. To understand the approach, it helps to begin by considering the equation used as the first stage of the 2SLS procedure:


(3) $$\begin{array}{l} \Delta U_{it}=\pi_{0}+\pi_{1}AH_{it}+\pi_{2}AH_{it}\times ITW_{i}+\mu_{i}+\lambda_{t}+\nu_{it}, \end{array}$$

This equation regresses abnormal unemployment on state- and week-level fixed effects (μ and λ), the stay-at-home rate (AH), and the IV, i.e., the interaction between stay-at-home rates and inverse teleworkability (*AH*×*ITW*).^10^ This regression's estimates are used to generate a prediction $\hat{\Delta U}$, which is then inserted into the second stage regression (Equation 2) as a replacement for Δ*U*.

Adopting the interaction term *AH*×*ITW* as IV is premised on the idea that, after being shocked with a certain amount of decreased labor mobility, relatively less teleworkable states may have experienced more abnormal unemployment than more teleworkable states. The crucial observation is that, since this difference would stem from structural conditions of each state's labor market, it is plausibly uncorrelated with the error term ϵ of Equation (2).^11^

Formally, our approach rests on the assumption that, conditional on the controls, AH × ITW affects statin demand only through abnormal unemployment. We think that this is a reasonable assumption, for the following reasons. First, the rich set of fixed effects and mobility series included in our specifications control for (i) state-specific time-invariant factors, (ii) nation-wide time-varying factors, and (iii) state-specific time varying factors pegged to mobility (AH). Second, when considering the leading channels (other than unemployment) by which AH × ITW could affect statin demand, there are two leading hypotheses. A first possibility pertains to reduced access to medical appointments, which are the source for the prescriptions needed to purchase products. However, contrary to this hypothesis, our estimate for the effect of unemployment on statin sales remain largely unchanged when we restrict attention to refill purchases, which do not require the issuance of a new prescription. A second possibility is that, given that our model does not include a price control, AH × ITW affects statin demand indirectly *via* price changes. Using the limited price information available from the sample, we find that price changes were largely independent of AH × ITW.

**Figure 3:**
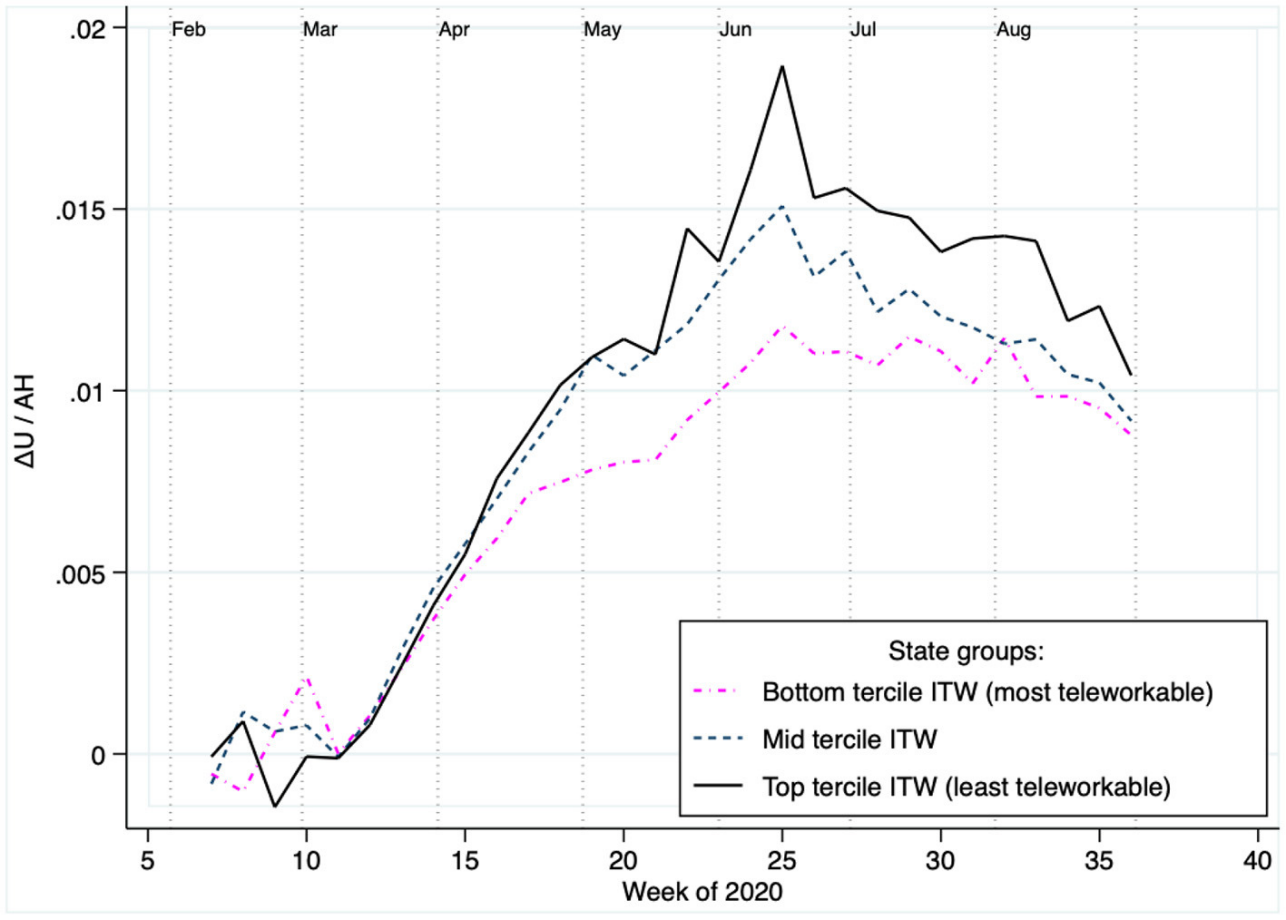
The teleworkability channel: Those states with higher teleworkability exhibited less abnormal unemployment per point of mobility reduction. Plots the amount of abnormal 2020 unemployment per point of abnormal mobility reduction (stay-at-home), aggregated across groups of states based on their teleworkability levels. The figure's main pattern indicates that in states with higher teleworkability there was less abnormal unemployment per point of mobility reduction.

Figure 3 presents evidence that is strongly consistent with the presence of the conjectured teleworkability channel for abnormal unemployment. Specifically, the figure presents series of mobility-adjusted abnormal unemployment (Δ*U*/*AH*) across groups of states of different teleworkability levels. That is, each curve represents the amount of abnormal unemployment per point of mobility reduction. The tercile of states of higher teleworkability levels (pink line) observed systematically lower abnormal unemployment per point of reduced mobility compared to those in the mid tercile (gray line), which in turn had systematically lower levels than those in the tercile of lowest teleworkability (black line). That is, holding constant mobility, more teleworkable states experienced less abnormal unemployment than less teleworkable ones. Econometric analyses presented below provide formal support for this result.

## 5. Results

### 5.1. OLS estimates

We start by reporting OLS parameter estimates. Specifically, in Table 4A we report the parameters obtained by separately estimating (Equation 2) on the data sub-samples associated with each of the four different payment modes (Cash, Medicaid, Medicare Part D, Third Party).

**Table 4:** OLS results.

**(1)**		**(2)**	**(3)**	**(4)**
**Cash**		**Medicaid**	**Medicare Part D**	**Third party**
**(A) All ages**				
Δ*U*	−0.5535^*^	0.1989	0.1877	0.1814
	(0.3142)	(0.2523)	(0.3884)	(0.2816)
AH	0.0013	−0.0032	0.0047	0.0019
	(0.0046)	(0.0036)	(0.0040)	(0.0047)
N	30,600	30,600	30,600	30,600
**(B)** < 64				
Δ*U*	−0.1399	0.1328	0.0796	−0.0924
	(0.3115)	(0.2827)	(0.2327)	(0.3560)
AH	−0.0003	0.0008	0.0046	−0.0041
	(0.0041)	(0.0041)	(0.0049)	(0.0059)
N	29,070	29,070	29,070	29,070
**(C)** 65+				
Δ*U*	−0.4860	0.2808	−0.0170	0.0634
	(0.3018)	(0.2351)	(0.3969)	(0.3167)
AH	0.0025	−0.0055	0.0047	0.0015
	(0.0050)	(0.0044)	(0.0045)	(0.0040)
N	30,600	30,600	30,600	30,600

All specifications include product, state, and week fixed effects. Robust standard errors (clustered at the state level) are displayed in parentheses. ^*^p < 0.1, ^**^p < 0.05, and ^***^p < 0.01.

Column 1 shows the estimates from the Cash payment sub-sample. The −0.5535 estimate for Δ*U*, which is significant at the 10% confidence level, associates one additional point of abnormal unemployment (Δ*U* = 0.01) with about 0.05% lower statin demand. Evaluated at the average of Δ*U* between mid March and August (i.e., during the pandemic lockdowns), at about 0.09, the effect amounts to an average 7% drop of statin sales. Contrary to this result, in the remaining sub-samples (Columns 2–4) we obtain estimates which associate abnormal unemployment with higher statin demand. Nevertheless, these estimates are of smaller magnitude than in Column 1, and are statistically insignificant as well. As such, with the exception to the Cash sub-sample, these results indicate a zero effect of unemployment on statin demand.

Table 4A also reports parameter estimates for the mobility variable (AH), which are positive in most cases. As such, they associate less mobility with more statin demand. At face value, these results are counter intuitive because we would expect decreased mobility to difficult trips to the pharmacy. These results can be reconciled by noting that all models include week-specific fixed effects, which capture the majority of the first-order effects of decreased mobility.^12^

Tables 4B, C repeat the analyses, first using the transactions made by people less than 65 years of age (Panel B), then using that of people of 65 and more.^13^ These results are qualitatively similar to those of Panel A, in that they provide little support for an unemployment effect on statin sales.

As noted in Section 4.2, the OLS estimate for the effect of abnormal unemployment on statin sales may be biased due to the presence of correlated omitted variables. A leading type of such confounder would correspond to variables reflecting the strength of the (multidimensional) public health response to the pandemic that was implemented by each state. To investigate this concern, we turned to the Commonwealth Fund's Scorecard on State Health System Performance, which provides a ranking for how well each state handled the COVID-19 pandemic from a public health standpoint.^14^ We approached these data with the idea that the Commonwealth Fund rankings act as a proxy for the potentially biasing confounds that motivate the concern. In particular, we expect that, in lower-ranking states (less robust public health response to COVID-19), spikes of abnormal unemployment will have “hit harder” in ways that are unobservable to Equation (2). As a result, if we estimated this equation state-by-state, we should obtain smaller (more negative) estimates for the effect of unemployment on statin sales among states that the Commonwealth Fund ranks as worse-performing against COVID-19. The results of our analysis were consistent with this idea. Specifically, we found that the state-level OLS effect of unemployment on statin sales (softly) decreases (becomes more negative) for states with lower positions in the Commonwealth Fund's ranking (see Figure A.1).^15^ As such, this analysis reaffirms the idea that OLS estimates may include an attenuation bias due to the presence of correlated omitted variables.

### 5.2. 2SLS estimates

#### 5.2.1. First stage

Table 5 presents the result of the first stage regression (Equation 3). Recall that this equation regresses abnormal unemployment (Δ*U*) on a series of controls and the IV (*AH*×*ITW*). Also recall that the stand-alone inverse teleworkability variable (*ITW*) is excluded from the model due to the presence of state-level fixed effects.

**Table 5:** First stage 2SLS results.

AH	0.0034^***^
	(0.0009)
AH × ITW	0.0037^***^
	(0.0012)
N	1,530
F	13.29

Estimates for Equation (3), where the dependent variable is abnormal unemployment (Δ*U*). The specification includes state and week fixed effects. Robust standard errors (clustered at the state level) are displayed in parentheses. ^*^p < 0.1, ^**^p < 0.05, and ^***^p < 0.01.

Consistent with the patterns of Figure 1, the estimate for *AH* associates less mobility (higher AH) with a larger amount of abnormal unemployment. The parameter estimate however does not lend itself for interpretation, as much of the mobility effects across the sample period are captured by the model's week-level fixed effects.

The table's main estimate is that for the IV (*AH*×*ITW*), which is positive and statistically significant with 99% confidence. Consistent with Figure 3, this estimate associates mobility reductions in less teleworkable states with larger increases of abnormal unemployment. For instance, the 0.0037 estimate associates one additional point of reduced mobility with over twice as much abnormal unemployment in the least teleworkable state (Wyoming), as compared to where teleworkability is at its maximum (Washington DC). Lastly, it is also important to note that the F statistic of 13.29 satisfies the commonly used rule of thumb of *F*>10 for weak instruments (34, 35).

#### 5.2.2. Second stage

The second stage results are presented in Table 6A. In three out of the four columns (Cash, Medicaid, Third Party), the parameter estimate for Δ*U* are smaller compared to their OLS counterparts. This feature of the results is consistent with the idea that, for these populations, the rise of COVID-fueled abnormal unemployment may have also prompted greater supplemental relief efforts.

**Table 6:** Second stage 2SLS results.

**(1)**		**(2)**	**(3)**	**(4)**
**Cash**		**Medicaid**	**Medicare Part D**	**Third party**
**(A) All ages**				
Δ*U*	−1.0191	−3.9549^**^	2.2193	−0.2050
	(1.5847)	(1.6277)	(1.9977)	(1.4636)
AH	0.0036	0.0173^**^	−0.0053	0.0039
	(0.0086)	(0.0077)	(0.0088)	(0.0079)
N	30,600	30,600	30,600	30,600
**(B)** < 64				
Δ*U*	−1.2856	−5.6419^**^	−0.6493	−2.7580
	(1.1752)	(2.4029)	(1.4588)	(2.1380)
AH	0.0053	0.0294^**^	0.0082	0.0090
	(0.0050)	(0.0123)	(0.0091)	(0.0114)
N	29,070	29,070	29,070	29,070
**(C)** 65+				
Δ*U*	−1.8541	1.8691	2.5536	−1.2560
	(2.4092)	(1.9200)	(2.3157)	(1.3838)
AH	0.0093	−0.0133	−0.0080	0.0080
	(0.0124)	(0.0098)	(0.0103)	(0.0053)
N	30,600	30,600	30,600	30,600

Estimates for Equation (2), where the dependent variable is abnormal sales (Δ*Q*) and the abnormal unemployment regressor is replaced its first stage prediction from Equation (3), $\hat{\Delta U}$. All specifications include product, state, and week fixed effects. Robust standard errors (clustered at the state level) are displayed in parentheses. ^*^p < 0.1, ^**^p < 0.05, and ^***^p < 0.01.

According to the estimates, the effect of unemployment on statin sales is only significant (with 95% confidence) among Medicaid transactions. Based on the parameter estimate of −3.95, one point of abnormal unemployment (Δ*U* = 0.01) leads to an about 4% drop of statin demand. Evaluated at the average of Δ*U* between mid March and August (i.e., during the lockdowns), about 0.09, the effect amounts to an average 31% drop of statin sales.

Table 6B repeats the analysis on the population aged less than 64 years old. Compared to in Panel A, all parameter estimates for abnormal unemployment are smaller, i.e., they point to a more pronounced negative unemployment effect on statin sales. This result is consistent with the idea that, given their working age, this population is more exposed to unemployment effects than those in the 65+ group. The parameter estimate for Medicaid transactions exhibits a noticeable increase of magnitude, down to −5.64 from −3.95 in Panel A. In this case, the estimate implies that one point of abnormal unemployment (Δ*U* = 0.01) leads to an almost 6% drop of statin demand. In turn, for the period between March and August of 2020, the implied effect amounts to a 40% drop of statin sales on average. Lastly, Panel C reproduces the estimates using data from the group aged 65 years or older. Within this population, estimated parameters do not point to any systematic effects.

### 5.3. Robustness checks

#### 5.3.1. Placebo test

**Figure 4:**
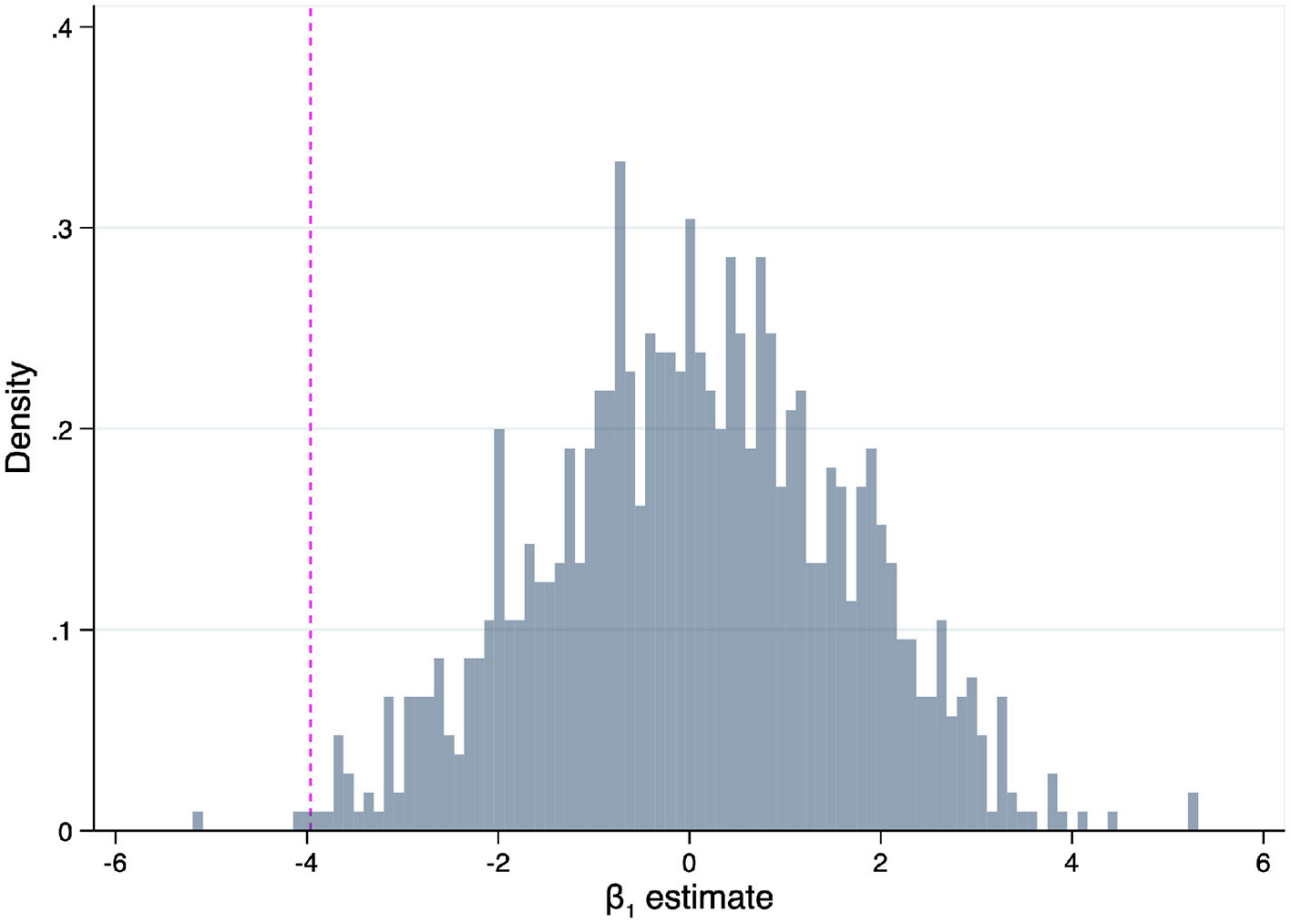
The falsification procedure randomly re-assigned Δ*Q* series across states (1,000 pseudo samples). The vertical line marks the 2SLS obtained from the non-falsified sample (Column 2, Table 6).

To probe the causal interpretation of our 2SLS estimate for the Medicaid sub-sample (Column 2, Table 6A), we conducted a placebo test. The test consisted of re-assigning a state's demand series (Δ*Q*) to a randomly chosen state. We repeated the 2SLS estimation procedure on each resulting pseudo-sample (*N* = 1,000). The distribution of β_1_ estimates, which is shown in Figure 4, resembles a normal distribution with mean 0 and standard deviation of approximately 1.5. Given this distribution, our β_1_ estimate for the Medicaid sub-sample (marked by the vertical line) is an event of very low probability (*p* = 0.002). We interpret these results as supportive for the estimate's causal interpretation.

#### 5.3.2. Access to prescribers

Is the negative effect of unemployment on Medicaid statin access solely attributable to patients' purchasing decisions? As an alternative, the effect may have been rooted on a contraction of medical appointments. That is, patients being unable to see a provider and thereby lacking the necessary prescription to make the statin purchase.

To investigate this possibility, we reproduced our analysis for Medicaid sales that only considers sales that represent refills. The rationale behind this test is that sales made via refills are far less dependent on access to medical appointments than total sales. This analysis resulted in a parameter estimate ${\hat{\beta }}_{1}=-3.6784$ (S.E. 1.8493), which is statistically significant at the 95% confidence level. This parameter estimate of similar magnitude compared to its analog obtained using total statin sales (Column 2, Table 6A). The similarity between the two estimates suggests that our main result is not driven by the influence of providers or access to medical visits.

#### 5.3.3. Price effects

As noted in Section 4, we were unable to include a price variable in our econometric specification due to missing price observations (in cases of zero sales). This omission introduces the worry that some of the effects documented above may be attributable to price changes. Evidence presented in this section suggests that this omission is unlikely to drive the estimated effects of unemployment on Medicaid statin sales.

We begin by noting that, to drive the reduction of statin sales that our estimates attribute to unemployment, omitted price variation should have taken a specific form. Specifically, abnormal price increases should have coincided with abnormal unemployment increases. To investigate whether this correlation is present in the data, we create a price index for Medicaid purchases. The index is defined at the state/week level, as:


(4) $${\bar{p}}_{it}=\sum_{j}s_{ijt}\cdot p_{ijt},\text{ with }s_{ijt}=\frac{Q_{ijt}}{\sum_{j^{\prime }}Q_{ijt}}.$$

This index addresses with the issue of missing data by aggregating across products. In doing so, the main computational element is *s*_*ijt*_, which corresponds to the share of product *j* among all doses sold (across products) in state *i* during week *t*, with $\underset{j}{\sum }s_{ijt}=1$ ∀ *i, t*. In turn, *p*_*ijt*_ corresponds to the average “customer price” variable, which is available from the IQVIA data for product *j* during week *t* (i.e., total sum of payments made to the pharmacy, from patient and insurance). In other words, the price index is constructed as a weighted average of observed prices, where more popular products weight more. Based on this definition, we construct


(5) $$\begin{array}{l} \Delta p_{it}=\log\left({\bar{p}}_{it}^{2020}\right)-\log\left({\bar{p}}_{it}^{2019}\right) \end{array}$$

in analogous fashion to previously-introduced variables.

In Table 7, we present the results of a series of regressions that use Δ*p* as dependent variable. The main regressor in the model of Column 1 is abnormal unemployment. (In addition to the listed regressors, all models of Table 7 include fixed state-level and week-level fixed effects.) The estimated parameter is negative, suggesting that Medicaid prices were inversely correlated with unemployment. This results suggests that, rather than doubling down on the effects of unemployment, price changes may have moderated them. However, notice that the parameter is estimated with a significant amount of error and is statistically insignificant.

**Table 7:** Assessing the exogeneity of medicaid prices.

	**(1)**	**(2)**	**(3)**
Δ*U*	−0.4391		−0.3588
	(0.4382)		(0.4784)
AH		−0.0094	−0.0082
		(0.0059)	(0.0056)
AH × ITW		0.0050	0.0063
		(0.0137)	(0.0141)
N	1,530	1,530	1,530

The dependent variable in all Column is Δ*p* (see Section 5.3.3 for details on its construction). All specifications include state and week fixed effects. Robust standard errors (clustered at the state level) are displayed in parentheses. ^*^p < 0.1, ^**^p < 0.05, and ^***^p < 0.01.

In Column 2, we focus on the formal IV assumptions, namely, that the IV is independent of the error term. We do so by regressing Δ*p* on the mobility measure (AH) and the IV, i.e., the interaction of mobility and tele-workability (AH × ITW). Both estimated coefficients are small and quite imprecise (statistically insignificant). The idea that abnormal price changes were independent of all key variables in our analysis is further reinforced by the regression of Column 3, which includes all regressors. Collectively, we take these results as strongly suggestive that price changes did not drive our result for Medicaid sales.

## 6. Discussion

Our findings suggest that, for individuals who rely on the Medicaid pharmacy benefits, the large abnormal unemployment rates caused by the COVID-19 pandemic may have led to diminished access to care for chronic medical conditions, specifically statin drugs.

Given that statin drugs play an essential role in the treatment and prevention of cardiovascular disease and are underutilized in the United States even among fully insured populations (27, 28), alternative strategies to achieve public health goals should be considered. Effective design of such strategies must consider the specific behavioral underpinnings of this phenomena. One alternative would be that the negative effect on statin access is caused by the financial implications of job loss. This scenario is supported by evidence pointing to copays as barriers to statin adherence (36). This channel is also supported by a series of reports linking decreased drug access due to financial strains attributable to COVID-related unemployment (14, 37). The channel is also consistent with the explicit recognition of several large manufacturers that, for those affected by COVID-related unemployment, important prescription drugs may have become less affordable during the pandemic (38).^16^ An alternative scenario would be that statin adherence is weakened because job separation inflicts other, more pressing problems on individuals, e.g., emotional distress. These issues prompt the individual to de-prioritize managing asymptomatic conditions, such as high cholesterol. Yet another possibility is that the increased time availability that results from job loss allows individuals to invest in desirable behaviors, like regular exercise and a healthy diet. The acquisition of these behaviors may induce a substitution effect, whereby patients conclude that statin adherence is of less importance. Whereas, price-based schemes such as coupons, discounts, or copay-waivers should receive primary consideration in the first scenario, they may be ineffective in the second and third cases.

Another important aspect of our findings pertains to health equity. Specifically, we observe that there exist persistent race- and gender-based disparities in labor market outcomes (39). The COVID-19 pandemic has amplified these disparities by disproportionately increasing unemployment among women and racial minorities (40–43). This observation suggests that the documented reductions of statin access may also be unequal along these dimensions. This consideration is of particular concern, given that statins were particularly underutilized by women and racial/ethnic minorities in the United States, even prior to COVID-19 (44).

## 7. Conclusion

Beyond the large number of deaths by infection, the COVID-19 pandemic has impacted the health of the American population by disrupting access to health care. These employment disruptions and the corresponding worsening of health outcomes have been exacerbated by the economic recession triggered by the pandemic, which had fueled unemployment levels beyond the levels observed during the great recession. This article evaluated whether there existed a systematic relationship between COVID-fueled unemployment levels across the United States and access to statin drugs during the initial phase of the pandemic. The analysis focused on statin medications—an WHO essential medicine, which are central for the treatment of cardiovascular disease. Our findings suggest that, while COVID-fueled unemployment has not hindered access to these medications for the majority of U.S. population, it has had a strong negative for a disadvantage minority (Medicaid insured).

## Data availability statement

The original contributions presented in the study are included in the article/Supplementary material, further inquiries can be directed to the corresponding author.

## Author contributions

MH, CA, and DP conceived of the presented idea and provided critical feedback and helped shape the research, analysis, and manuscript. MH compiled the datasets, designed and executed the empirical analysis, and took the lead in writing the manuscript. All authors contributed to the article and approved the submitted version.

## Footnotes

^1^See https://www.nytimes.com/interactive/2021/us/covid-cases.html.

^2^Consistent with these findings, the availability of unemployment insurance have been associated with relatively higher healthcare among beneficiaries, lower risk for access delays and unmet health-related needs, and better mental health outcomes (14, 15).

^3^To systematize states' unemployment vulnerability levels to mobility restrictions, we rely on the framework of Dingel and Neiman (16) and Mongey et al. (17).

^4^A fifth modality, Medicare, accounts for a very minor share of all dispensed doses (0.03%). We append these transactions to the Medicare Part D modality.

^5^See https://www.onetonline.org/help/online/data.

^6^From the work context module, we consider questions on the importance of: (1) Email use, (2) Outdoors work, (3) Dealing with violent people at least once a week, (4) Wearing common or specialized protective or safety equipment, (5) Walking or running, (6) Exposure to minor burns, cuts, bites, or stings, and (7) Exposure to diseases or infection. From the work activities module, we consider questions on the importance of: (1) General Physical Activities, (2) Handling and Moving Objects, (3) Controlling Machines and Processes (not computers nor vehicles), (4) Operating Vehicles, Mechanized Devices, or Equipment, (5) Performing for or Working Directly with the Public, (6) Repairing and Maintaining Mechanical Equipment, (7) Repairing and Maintaining Electronic Equipment, and (8) Inspecting Equipment, Structures, or Materials.

^7^See https://www.bls.gov/oes/tables.htm.

^8^For illustrative purposes, the data plotted by Figure 2B aggregates occupations at the 2-digit SOC code level. However, to compute the ITW score (Equation 1), occupations are defined at the 3-digit SOC code level.

^9^We treat Washington DC as a state because all data sources treat it as such.

^10^Equation (3) omits the stand-alone ITW variable because it would be redundant given the inclusion of state-level fixed effects.

^11^This approach (IV as an interacted variable) has been used by several other recent works, e.g., Dube and Vargas (30), Hanna and Oliva (31), Nunn and Qian (32), Peri (33).

^12^The full-sample correlation between Δ*Q* and AH is negative (ρ = −0.0052, *p* = 0.0707).

^13^Given the small share of transactions associated with the “ < 40” group, we have combined their data with the 40-64 group a broader “ < 64” group.

^14^See https://www.commonwealthfund.org/publications/scorecard/2022/jun/2022-scorecard-state-health-system-performance.

^15^This evidence is based on the sample of Medicaid transactions. We focused on this sample because this is the only segment where our results in the next section unveil causal effects of unemployment on statin sales.

^16^For example, during this period, Eli Lilly offered their insulin at reduced prices. The president of the company's diabetes branch stated: “Too many people in the U.S. have lost their jobs because of the COVID-19 crisis, and we want to make sure no one goes without their Lilly insulin” (38).

## Author disclaimer

The statements, findings, conclusions, views, and opinions contained and expressed in this article are based in part on data obtained under license from the following IQVIA Incorporated information service(s), All Rights Reserved. The statements, findings, conclusions, views, and opinions contained and expressed herein are not necessarily those of IQVIA Incorporated or any of its affiliated or subsidiary entities.
