# Supplementary material for: COVID-19 unemployment and access to statin medications in the United States
Source: Front Public Health. 2023 Mar 30;11:1124151. doi: 10.3389/fpubh.2023.1124151 (PMC10097886; doi:10.3389/fpubh.2023.1124151)
Supplement: Supplementary file 1 [file Data_Sheet_1.pdf]

Supplementary Material

"Covid-19 Unemployment and Access to Statin Medications in the United States"

Figure A.1: Illustrating Potential OLS Bias: Evidence from the Commonwealth Fund's Ranking on State-level Public Health Performance Against the Covid-19 Pandemic.

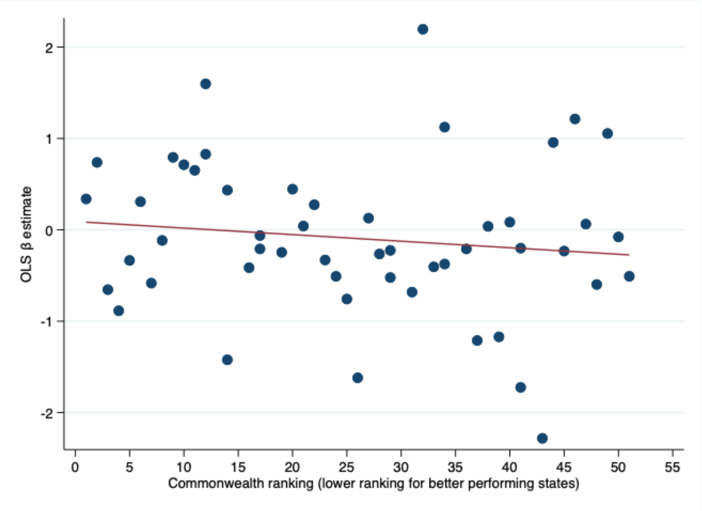

Notes. The vertical axis plots OLS β parameters obtained by separately estimating Equation 2 on the sample of Medicaid transactions of each state (i.e., state-level version of the estimate of Column 2, Panel A, Table 4). The horizontal axis plots the Commonwealth Fund's ranking for each state's public health response against the Covid-19 pandemic (see `https://www.commonwealthfund.org/publications/scorecard/2022/jun/2022-scorecard-state-health-system-performance`). The best fitting line (in red) represents a correlation of $\rho = -0.13$.

Table A.1: Inverse Teleworkability (ITW) by State.

State	ITW
District of Columbia	0.0000
Massachusetts	0.4749
New York	0.4761
Connecticut	0.5093
Maryland	0.5834
Rhode Island	0.5846
New Hampshire	0.5923
Delaware	0.5933
Arizona	0.6005
New Jersey	0.6034
Virginia	0.6042
Vermont	0.6054
California	0.6129
Minnesota	0.6131
Colorado	0.6211
Utah	0.6323
Illinois	0.6347
Puerto Rico	0.6364
Pennsylvania	0.6582
Washington	0.6629
Missouri	0.6724
Oregon	0.6751
Florida	0.6751
Texas	0.6940
Ohio	0.7026
Guam	0.7061
Georgia	0.7121
Michigan	0.7150
Kansas	0.7170
Maine	0.7173
Nebraska	0.7189
Alaska	0.7201
North Carolina	0.7252
Oklahoma	0.7357
Wisconsin	0.7373
New Mexico	0.7394
South Dakota	0.7399
Iowa	0.7417
Idaho	0.7418
Montana	0.7429
Hawaii	0.7497
Tennessee	0.7661
Virgin Islands	0.7707
South Carolina	0.7805
Nevada	0.7858
Kentucky	0.7993
Alabama	0.8182
Arkansas	0.8243
Indiana	0.8325
West Virginia	0.8385
Mississippi	0.8428
North Dakota	0.8756
Louisiana	0.8773
Wyoming	1.0000
